# Predicting factors and prevalence of meningitis in patients with first seizure and fever aged 6 to 18 months

**Published:** 2014-10

**Authors:** Farhad Heydarian, Farah Ashrafzadeh, Atefeh Rostazadeh

**Affiliations:** *From the Department of Pediatrics, Ghaem Hospital, Mashhad, Iran*

## Abstract

**Objective:**

To evaluate predicting factors and prevalence of meningitis in patients with first seizure and fever aged 6-18 months old.

**Methods:**

This cross-sectional study was performed on 800 patients aged 6-18 months old who had first attack of seizure with fever between March 2005 and March 2012 in the pediatric ward of Ghaem Hospital, Mashhad, Iran.

**Results:**

Among 800 patients, lumbar puncture (LP) was performed in 453 (56.6%) patients, of whom 80 cases had meningitis (17.6% of LP patients). Postictal drowsiness (*p*=0.003), neurologic deficit (*p*=0.000), and body temperature ≥38.5°C (*p*=0.035) were among the clinical signs, which were statistically significant predicting factors for meningitis. Laboratory tests including white blood count (WBC) ≥15000 mm^3^ (*p*=0.004), and hemoglobin (Hb) <10.5 gr/dl (*p*=0.020) also had statistical significance in predicting meningitis.

**Conclusion:**

Postictal drowsiness, neurological deficit, body temperature ≥38°5C, WBC ≥15000 mm^3^, and Hb <10.5 gr/dl were clinical and laboratory factors predictive of meningitis in cases with first attack of seizure and fever.

Febrile seizure (FS) is the most common convulsive disorder in children with some probable risk factors that occur in 2-4% of children aged 6 months to 5 years old. It is defined as a seizure attack due to rising body temperature above 38°C in the absence of metabolic disease or CNS infection. It is divided into the simple form, which is single, less than 15 minutes with generalized seizure, and the others, known as the complex form.^1^-^6^ Seizure can be seen in meningitis in one out of 6 patients of whom one-third have no signs of meningeal involvement.^7^ Some predicting factors of meningitis in young children who present with seizure and fever may be seizure duration above 30 minutes, neurologic deficit, and postictal drowsiness.^8^ Prevalence of meningitis ranges between 2.4-17% in those children who have seizure and fever.^8^-^10^ In view of the paucity of previous studies, we undertook this study to evaluate the predicting factors and prevalence of meningitis in our patients with first attack of seizure with fever.

## Methods

This cross-sectional study was performed on 800 patients aged 6-18 months old who had first attack of seizure with fever between March 2005 and March 2012 in the Pediatric Ward of Ghaem Hospital, Mashhad, Iran. Patients were divided into 2 groups. Those with simple FS with one tonic colonic seizure attack for less than 15 minutes. Those cases who had multiple attacks or seizures lasting more than 15 minutes, or had focal seizures were categorized as complex FS.

The patient records were evaluated for LP for meningitis, which was defined as abnormal changes in cell count, protein, and sugar as well as positive culture result. Age, gender, seizure type and duration, postictal drowsiness, neurologic deficit, body temperature, previous family history of FS, white blood cell count (WBC), and hemoglobin (Hb) were also recorded.

The inclusion criteria included patients aged 6-18 months old with first attack of convulsion and fever. Exclusion criteria included any history of FS or epilepsy, any neurological disorder including hydrocephaly, cerebral palsy, any metabolic disorder including hypoglycemia, hypocalcemia, hypomagnesemia, traumatized LP, and incomplete records. All cases that met the inclusion criteria were included. The Ethics Committee of Mashhad University of Medical Sciences approved the study, which was carried out according to the Principles of the Helsinki Declaration.

A statistician calculated the sample size, and data were analyzed using the Statistical Package for Social Sciences version 11 (SPSS Inc., Chicago, IL, USA). Statistical tests including chi square and t test were used. Logistic regression analysis was used to evaluate predicting factors for meningitis. Statistical significance was set at a p-value <0.05.

## Results

The mean age of patients was 12.76 months (standard deviation [SD] 3.98). Most of the cases (435 [54.4%]) were male. Among 800 patients, LP was performed in 453 (56.6%), of whom 80 cases had meningitis (17.6% of LP patients). Bacterial meningitis was detected in 5 (6.25%) including one pneumococcal, 3 Hemophilus influenza type b (Hib), and one enterococcus. A comparison of some of the clinical aspects of patients with and without meningitis is shown in **Table 1**.

**Table 1:** Clinical aspects of first seizure patients aged 6-18 months with and without meningitis that underwent lumbar puncture.

Variable	Meningitis, n (%)		*P*-value
	Positive (N=80)	Negative (N=373)	
***Gender***			0.90
Male	42 (52.5)	193 (51.7)	
Female	38 (47.5)	180 (48.3)	
***Febrile seizure***			0.83
Apparently simple	35 (43.8)	168 (45.0)	
Apparently complex	45 (56.2)	205 (55.0)	
***Frequency of seizure attack***			0.61
Once	55 (68.8)	267 (71.6)	
Twice or more	25 (31.2)	106 (28.4)	
***Duration of seizure***			0.15
<15 mins	74 (92.5)	323 (86.6)	
≥ 15 mins	6 (7.5)	50 (13.4)	
***Postictal drowsiness***			0.003
Yes	69 (86.2)	260 (69.7)	
No	11 (13.8)	113 (30.3)	
***Neurological deficit***			0.000
Yes	4 (5.0)	1 (0.3)	
No	76 (95.0)	372 (99.7)	
***Body temperature***			0.035
<38.5°c	38 (47.5)	225 (60.3)	
≥38.5°c	42 (52.5)	148 (39.7)	
***Hemoglobin***			0.02
<10.5 _gr/dl_	49 (61.2)	175 (46.9)	
≥10.5 _gr/dl_	31 (38.8)	198 (53.1)	
***White blood cell count***			0.004
<15000 mm^3^	50 (62.5)	291 (78.0)	
≥15000 mm^3^	30 (37.5)	82 (22.0)	
***Family history of febrile seizure***			0.024
Yes	6 (7.5)	66 (17.7)	
No	74 (92.5)	307 (82.3)	
***Time of seizure occurrence from beginning of fever***			0.38
<24 hours	36 (45)	188 (50.4)	
>24 hours	44 (55)	185 (49.6)	

According to logistic regression analysis, independent variables for predicting meningitis were neurological deficits, postictal drowsiness, body temperature, level of Hb, and WBC (**Table 2**). The risk of meningitis was 30 times greater in cases who had neurological deficits than in those without. It was 3 times greater in those with postictal drowsiness, and 1.7 times greater in those with fever ≥38.5°C. The risk of meningitis was 2.3 times greater in cases with a WBC ≥15000 mm³, and 2 times greater in cases with Hb <10.5 gr/dl.

**Table 2:** Independent variables for predicting meningitis among first seizure patients aged 6-18 months with and without meningitis.

Variables	EXP(B)	95% CI lower-upper	*P*-value
***Neurologic deficit***		2.741-322.476	0.005
Yes	29.731		
No	1		
***Postictal drowsiness***		1.581-6.433	0.001
Yes	3.189		
No	1		
***Body temperature***		1.033-2.887	0.037
≥38.5°C	1.727		
<38.5°C	1		
***White blood cell count***		1.368-4.072	0.002
≥15000 mm^3^	2.360		
<15000 mm^3^	1		
***Hemoglobin***		1.156-3.293	0. 012
<10.5 gr/dl	1.951		
≥10.5 gr/dl	1		

EXP (B) - exponent (base), 95% CI - 95% confidence interval

## Discussion

We found that postictal drowsiness, neurological deficit, body temperature ≥38.5°C, WBC ≥15000 mm^3^, and Hb <10.5 gr/dl are among predicting factors for meningitis in cases admitted for evaluating fever and seizure.

Batra et al^8^ performed a study on children admitted with first attack of seizure and fever, and lumbar puncture was performed in 199 (40%) of cases. They detected that postictal drowsiness, duration of convulsion >30 minutes, and neurological deficit were among predicting factors for meningitis. However, apparent complex febrile seizure, leukocyte count ≥15000 mm^3^, and Hb level were not statistically significant between the meningitis and non-meningitis group. In another study by Joshi Batajoo et al^10^ on children admitted for first attack of seizure and fever, meningitis was revealed in 17% of cases. They detected that 4.5% had bacterial meningitis in patients aged 6-12 month, and the meningitis prevalence was higher in younger children.

Ghotbi et al^9^ also conducted a study on children with first attack of seizure and fever, and LP was carried out in all patients. They found that age <one year, complex FS, previous antibiotic usage, drowsiness and signs of meningeal involvement were statistically significant in cases who had meningitis.

In another study by Tinsa et al,^11^ which was performed on infants, patients were divided into 2 groups, those with febrile convulsive, and those with seizure due to meningitis. Their results revealed that predicting factors for meningitis were seizure lasting more than 5 minutes, age <7 months, neurological abnormality, and partial seizure.

Chin et al^12^ performed a study in London on patients aged 29 days to 15-years-old. They found that 17% of cases of status epilepticus associated with fever had acute bacterial meningitis. They also detected that 1.2% of patients with acute bacterial meningitis had short seizure. Accordingly prolonged seizure can be an important sign of meningitis in patients with seizure and fever.

In a study by Kimia et al^13^ on 704 cases aged 6 months to 18 months with first simple febrile seizure, LP was performed in 271 (38%) cases. Their study revealed that 10 cases (3.8%) had aseptic meningitis, with CSF abnormal cell count and no bacterial infection. In that study, they found that acute bacterial meningitis is rare in patients with first simple febrile seizure. In another study by Kimia et al^14^ carried out on children with first complex FS, LP was carried on in 340 cases (64%). They detected 3 cases (0.9%) of acute bacterial meningitis. Again indicating that acute bacterial meningitis is very low in patients with first complex febrile seizure. In our study, acute bacterial meningitis was detected at a higher rate (6.25%) compared with other studies.

The differences in prevalence of meningitis between the current work and previous studies (**Table 3**) can be partially related to differences in the age of patients, and the vaccination status, including the very effective conjugate pneumococcus or Hib vaccines, which was available to patients in Kimia et al’s studies.^13^,^14^ There is no current routine vaccination program for pneumococcus and Hib in Iran). Geographic, cultural, and ethnic differences are also factors. Parents may not always permit LP, and sometimes the LP is not successful.

**Table 3:** Rate of meningitis in children with seizure and fever from different studies.

Study	Number of cases	Age (months)	Meningitis (%)
Batra et al, India^8^	497	6-18	2.4
Joshi Batajoo et al, Nepal^10^	175	6-60	17.0
Ghotbi & Shiva, Iran^9^	254	6-60	4.7
Tinsa et al, Tunis^11^	106	<12	10.0
Kimia et al USA^14^	526	6-18	2.7
Current study	453	6-18	17.6

A limitation of the current study was lack of follow up of those cases that did not undergo LP due to admittance to another hospital. Further study of these patients is required.

In conclusion, we found a higher prevalence of meningitis in our study (17.6% in LP patients), of whom 6.25% had bacterial meningitis. We found clinical and laboratory factors that predict meningitis in cases with first attack of seizure and fever including; postictal drowsiness, neurological deficit, body temperature >38°5C, WBC ≥15000mm^3^, and Hb <10.5gr/dl.
